# Retinal whole genome microarray analysis and early morphological changes in the optic nerves of monkeys after an intraorbital nerve irradiated injury

**Published:** 2011-11-15

**Authors:** Yong Xia, Jun Chen, Li Xiong, Jiagang Liu, Xuesong Liu, Lu Ma, Qiao Zhang, Chao You, Jing Chen, Xuyang Liu, Xiaoyu Wang, Yan Ju

**Affiliations:** 1 Department of Neurosurgery, West China Hospital, Sichuan University, Chengdu, Sichuan Province, P.R. China; 2 Department of Ophthalmology, West China Hospital, Sichuan University, Chengdu, Sichuan Province, P.R. China

## Abstract

**Purpose:**

To obtain and analyze early retinal changes at the molecular level 24 h after a radiation injury to the ipsilateral intraorbital nerve using gamma knife surgery (GKS), and to examine the morphological changes in bilateral optic nerves.

**Methods:**

Unilateral intraorbital optic nerves of three *rhesus macaques* were treated by GKS with irradiated doses of 15 Gy, while contralateral optic nerves and retinas served as the control. Gene expression profiles of the control and affected retinas were analyzed with Affymetrix Rhesus Macaque Genome arrays. To verify the results, a quantitative real-time polymerase chain reaction (qRT–PCR) was performed to test the expression patterns of five function-known genes. Morphological changes in the bilateral optic nerves were examined using a transmission electron microscope (TEM) and light microscopy. The glial cell reaction in bilateral optic nerves was studied using immunohistochemistry.

**Results:**

Of the probe sets, 1,597 (representing 1,081 genes) met the criteria for differential expression, of which 82 genes were significantly up-or down-regulated in treated retinas. There was prominent upregulation of genes associated with glial cell activation in the treated retina. Genes related to an early inflammatory reaction and to cell death were also significantly regulated in response to a radiation injury to the intraorbital optic nerve. In contrast, the messenger ribonucleic acid (mRNA) expression levels of retinal ganglion cell (RGC)-specific genes were low. Morphologically, cytoplasmic processes of astrocytes in treated nerves were shorter than those of the control and were not straight, while also being accompanied by decreased GFAP immunostaining. More oligodendrocytes and inflammatory cells were apparent in treated nerves than in the control. In addition, swollen mitochondria and slight chromation condensation could be seen in the glial cells of treated nerves.

**Conclusions:**

We conclude that the current irradiated dose of 15 Gy was sufficient to lead to a radiation injury of the optic nerve and retina. Several transcripts deregulated in retinas after a radiation injury play a key role in radiation-induced neurogenic visual loss, especially for genes associated with RGC, glial cell, and cell death. Glial cells in optic nerves might be the primary target of a radiation injury in the optic nerve.

## Introduction

Tumors involving or adjacent to anterior visual pathways are common in ophthalmology and neurosurgery. Several studies demonstrate that complete removal of these tumors by enucleation in critical locations, such as the optic nerve sheath, optic canal, or superior orbital fissure, may result in direct injury or vascular impairment to the optic apparatus and metastatic spread [1], followed by vision loss [2]. Therefore, tumor control with organ preservation and the prevention of metastasis are the most important goals of the treatment [1]. Gamma knife surgery (GKS) is currently one of the most precise radiotherapy techniques in stereotactic radiation therapy, having not only the advantage of being minimally invasive, but also allowing highly conformal dose distribution with a steep dose fall-off. That is to say, stereotactic radiosurgery is a suitable selection for orbital lesions, and in fact, many reports have yielded promising results [1,3-5]. However, the tissues of the anterior visual pathway significantly differ in terms of their molecular makeup, cell populations, and their response to ionizing radiation from other brain tissues, while being more sensitive than other cranial nerves. During radiation therapy, the radiation dose that could kill or control the growth of tumors may lead to complications, including visual field defects, irreversible visual loss, and even zero light perception. The precise mechanism for radiation injury is yet to be determined.

Injury to the optic nerve will lead to a programmed set of immediate and early response gene deregulations in the retina [5-7]. Severe radiation injuries to the optic nerve can trigger retinal ganglion cell (RGC) death, resulting in visual field defects and visual loss [8]. In our study, we randomly treated the unilateral intraorbital nerve using a 15 Gy radiation dose with a 50% isodose curve. The mean dose for the contralateral optic nerve, optic chiasm, and retina was limited to less than 3 Gy by employing multiple small isocenters and using plugs. This has been shown to result in a radiation injury to the intraorbital nerve because the actuarial incidence of optic neuropathy for patients who received an irradiated dose of 15 Gy or more is 77.8% [9].

Given the complexity of radiation-induced responses, microarrays are useful tools for identifying a wider range of genes involved in the development of a radiation injury [10]. Many studies have reported correlations between gene expression and radiotherapeutic response [11-13], survival time after regrowth [12], radiation-induced resistance and tumorigenesis [14], and in vitro radiosensitivity [15-18]. Furthermore, Chinnaiyan [19] reported the largest cohort of differentially regulated genes to emerge 24 h after exposure to radiation. The present study focuses on the changes in gene expression in whole retinas, particularly the RGC, in the 24 h following an intraorbital nerve irradiated injury, with the aim of evaluating retinal roles in the process of radiation-induced visual loss after GKS, which includes significantly expressed genes related to radiation-induced cell death, glial cell reaction, and some key pathways. In addition to a microarray analysis, we also evaluated the roles and impact of morphological changes to bilateral optic nerves in this process using a TEM, light microscopy, and immunohistochemistry.

## Methods

### Animals

All experiments using rhesus monkeys were approved by the Institutional Review Board and followed the relevant ethical regulations. Three male monkeys—aged 8–10 years and with a mean bodyweight of 8 kg—were obtained from the PingAn Animal Breeding & Research Base (Chengdu, China). They were individually housed under a 12 h:12 h light-dark cycle (light intensity during the day was approximately 100 lx), in a quiet room without strong light, and with free access to water and food. They were cared for in accordance with NIH Guidelines for the Care and Use of Laboratory Animals (1996). Every effort was made to minimize the suffering of the animals.

### Gamma Knife treatment

Anesthesia was induced intramuscularly with ketamine (10 mg/kg, Bei Jing Shuang He Pharmaceutical LLC, Beijing, China) and was maintained by the continuous administration of pentobarbital (6–9 mg/kg/h, IV, Wu Han He Zhong Chemical manufacturing Co., LTD, Wuhan, China) using catheters. A Leksell G stereotactic head frame (Elekta Instruments AB, Stockholm, Sweden) was placed at the bilateral occipital bone and on either side of the frontal bone. Magnetic resonance imaging (MRI) was performed for target localization (T1-weighted and T2-weighted images, 1.5 mm-thick axial slices). The MRI positioning data were processed and transferred to the Leksell Gammaplan^®^ version 9.0 (Elekta Instruments AB, Stockholm, Sweden). Conformal dose planning was established using Leksell Gammaplan^®^ version 9.0 to localize the target point and the radiation dose. Target volumes and critical structures were delineated manually by the surgeon, using axial images with the simultaneous overlay of the outlines on coronal and sagittal images. The size of the collimator was 4 mm, and the target point was selected randomly at the unilateral intraorbital nerve. Treatments were performed using the Leksell Gamma Knife C (Elekta Instruments AB) and its automatic positioning system. We prescribed 15 Gy to the 50% isodose line. The GKS procedure was performed on only one optic nerve from each experimental monkey.

### Surgery

At 24 h post-irradiation, the six eyes and the six intraorbital nerves of the three monkeys were removed. The retinas without retina pigment epithelium were rapidly (<5 min) dissected from the eyeball on ice [20], according to the method of Glowinski and lversen (1966), but with minor modifications [21]. Each sample was immediately labeled then flash-frozen in liquid nitrogen and stored at −80 °C before further analysis. The temporal halves were used for RNA isolation, while nasal retinas were used for backup [22].

### Histopathological examination

According to the manufacturer’s instructions, immunohistochemical staining was performed on 4-μm sections of formalin-fixed, paraffin-embedded retrobulbar portions of each optic nerve (half as long as the intraorbital optic nerve) by antibodies to Oligodendrocyte lineage transcription factor2 (Olig2; gift of C. Stiles, Harvard Medical School, Boston, MA), glial fibrillary acidic protein (GFAP; Dako, M0761, Glostrup, Denmark, M0761), and human progenitor cell antigen (CD34; sc-7324, Santa Cruz Biotechnology, Santa Cruz, CA, sc-7324). For electron microscopy, the remaining half of each optic nerve was rapidly (<1 min) prefixed in a mixed solution of 4% paraformaldehyde and 2.5% glutaraldehyde with postfixation in osmium tetroxide (OsO_4_; Nacalai Tesque, Inc. Kyoto, Japan). The tissues were then gradually dehydrated in increasing concentrations of acetone and were finally embedded in Epon812 (Shell Chemical Co., San Francisco, CA). The semithin sections were stained with methylene blue and the ultrafine sections were enhanced with 4% lead/uranylnitrate. Sections were examined with a TEM (H-600IV; Hitachi, Osaka, Japan).

### Retinal tissue RNA extraction and microarray experiments

The retinas of three monkeys that were ipslateral to the irradiated optic nerve were defined as treated retinas (monkey #01 left retina, monkey #02 left retina, and monkey #03 right retina) and three contralateral retinas were control retinas (monkey #01 right retina, monkey #02 right retina, monkey #03 left retina). The total RNA of the six samples was extracted using TRIzol reagent (Invitrogen Crp., Carlsbad, CA) and according to the manufacturer’s instructions. The RNA was then amplified, labeled, and purified using a GeneChip 3′IVT Express Kit (Catalog number 901229; Affymetrix, Santa Clara, CA) and following the manufacturer’s instructions to obtain biotin-labeled cRNA targets for the Affymetrix GeneChip^®^ Rhesus Macaque Genome Array (Affymetrix), which contained 47,000 transcripts. Array hybridization and washing was performed using a GeneChip^®^ Hybridization, Wash, and Stain Kit (Catalog number 900720; Affymetrix) in a Hybridization Oven 645 (Catalog number 00–0331–220V; Affymetrix) and Fluidics Station 450 (Catalog number 00–0079; Affymetrix), following the manufacturer’s instructions. After hybridization, slides were scanned using the GeneChip^®^ Scanner 3000 (Catalog number 00–00212; Affymetrix) and Command Console Software 3.1 (Affymetrix) under the default settings. Raw data were normalized by a MAS 5.0 algorithm, Gene Spring Software 11.0 (Agilent Technologies, Santa Clara, CA). Genes related to RGC, glial cells, and cell death were selected for further analysis. A two-way clustering analysis was performed using Cluster 3.0 to define similar sample patterns and differentially expressed genes among three separate groups. Furthermore, the significant genes (p<0.05 and fold change >2) were grouped in functional categories based on the Gene Ontology database (GO) and functional pathways were also analyzed using the online Kyoto Encyclopedia of Genes and Genomes (database KEGG; GeneChip 3VT express kit, user manual).

### Statistical analysis

All values were presented as the mean±SD. Statistical analysis was performed using the paired Student's *t*-test, and p<0.05 was considered to represent a significant difference.

### Quantitative real-time polymerase chain reaction (qRT–PCR)

Primers for qRT–PCR were designed for selected genes using Primer Express 2.0 software (ABI, Applied Biosystem, Foster City, CA) and checked for homology with the monkey genome using Primer-BLAST to achieve a high probability of XX-specific products. Product specificity was examined using a melting curve analysis of the ABI 7500 FAST System (ABI, Applied Biosystem). Standard curves were performed through dilution of PCR amplicons in a reverse transcription buffer to measure primer pair efficiency. The qRT–PCR experiment was performed in triplicate.

Using separate retinas for confirmation by qPCR is preferred, but it is difficult to obtain separate tissues. Consequently, we used partial retinal tissues from each monkey for qRT–PCR. Briefly, a total of 0.5 μg of RNA from each sample was transcribed into cDNA using the PrimerScript RT reagent Kit (Takara Biochemicals, Tokyo, Japan) according to the manufacturer’s protocol. The RT product (10 μl) was diluted with H_2_O up to 100 μl. cDNA was amplified by PCR with primers specific to the target sequence. Amplification conditions were as follows: 15 min incubation at 37 °C, followed by a 5 s termination reaction at 85 °C. Dilutions of cDNA in the PCR were adjusted for each gene with the aim of staying within the linear range of amplification. qRT–PCR was performed with SYBR Green Realtime PCR Master Mix and ABI 7500 FAST for qRT–PCR and according to the manufacturer’s instructions. Cycling conditions were as follows: initial stage of 15 s at 95 °C, 40 cycles of melting (95 °C for 5 s), annealing and extending (62 °C for 34 s), and default of the dissociation stage. The genes studied, including beta-2 microglobulin (*B2M*), growth arrest and DNA-damage-inducible, beta (*GADD45B*), murine double minute (*MDM*), scrapie responsive gene one (*SCRG1*), brain-derived neurotrophic factor (*BDNF*), 18S rRNA (*18S*) were chosen based on a combination of the array results and the potential biologic importance of the gene in question. Expression values were normalized to those of the *18S* gene as described previously. Relative quantization was performed using the 2^-ΔΔCt^ method, including an efficiency correction. Primers used in qRT–PCR and their sequences are shown in Table 1. Following the PCR, the amplicon melting curve was checked for PCR specificity.

**Table 1:** Oligonucleotide primers used during real-time polymerase chain reaction.

**Gene**	**Forward primer (5′-3′)**	**Reverse primer (5′-3′)**
*B2M*	GAGTATGCCTGCCGTGTGAAC	GCGGCATCTTCAAACCTCC
*BDNF*	ACTCAGGCCGAATGATCAAGG	TGTCCTGGCCTCTTCCACTG
*GADD45B*	TCGGCCAAGTTGATGAATGTG	GCGTGAAGTGGATTTGCAGG
*MDM2*	TTGATGAAAGCCTGGCTCTGT	CACCAGCATCAAGATCCGG
*SCRG1*	TGGACATTGTTGGCATTGGTT	GCCTATGGCTCTTCATCTCGG
*18S* (eucaryon)	CGGCTACCACATCCAAGGAA	GCTGGAATTACCGCGGCT

## Results

**Figure 1:**
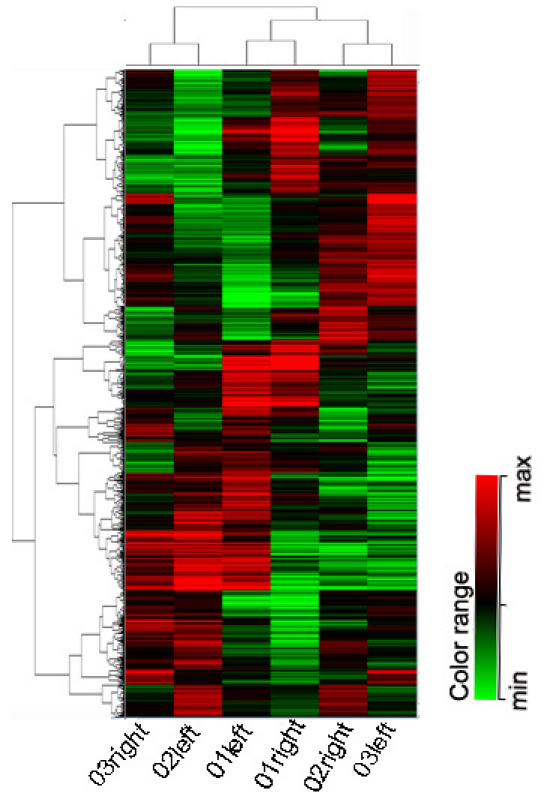
Coupled two-way clustering of samples and differentially expressed genes. A clustering analysis was performed to show the pattern of similarities among three separate groups after radiation treatment, using the differentially expressed genes. Rows represent genes, and columns represent samples. Red and green blocks, respectively, represent high and low expression relative to the control sample, and black blocks indicate equal expression, for which the standard deviation from the mean value is shown at the right. The cluster image shows the treated sides (monkey 01left retina, monkey 02left retina, monkey 03right retina) and the control sides (monkey 01right retina, monkey 02right retina, monkey 03left retina).

The experimental strategy for this project was to differentiate gene expression changes in treated retinas relative to the control retinas, as well as to examine the pathophysiological changes in bilateral optic nerves. After performing paired Student’s *t*-tests using the GeneSpring GX 11.0 software, 1,597 probe sets—representing 1,081 genes (656 up, 425 down)—and 516 expressed sequence tag (EST) clones met the criteria for differential expression (*t*-test, p<0.05). Part of the unclassified genes was hypothetical genes (527 genes) of unknown function. Among these different genes, our data revealed that RGC-specific genes, including basic helix–loop–helix family, member e40 (*BHLHE40*), centrosomal protein 97 kDa (*CEP97*), POU domain, class 4, transcription factor 2 (*POU4F2*), iroquois related homeobox 3 (*IRX3*), signal transducer and activator of transcription 1 (*STAT1*), tubulin, beta 3 (*TUBB3*), alcohol dehydrogenase 5 (class III), chi polypeptide (*ADH5*), microtubule associated monoxygenase, calponin and LIM domain containing 2 (*MICAL2*), guanine nucleotide binding protein, alpha stimulating complex locus (*GNAS*), sodium channel, voltage-gated, type I (*SCN1B*), FAT tumor suppressor homolog 3 (*FAT3*), tumor necrosis factor receptor superfamily, member 21 (*TNFRSF21*), tyrosine 3-monooxygenase/tryptophan 5-monooxygenase activation protein, eta polypeptide (*YWHAH*), nel-like, type 2 (*NELL2*), were deregulated in the treated retinas according to published microarray data [23-27] (Appendix 1). Genes associated with glial cell activation, including TIMP metallopeptidase inhibitor 1 (*TIMP1*), glial fibrillary acidic protein (*GFAP*), caveolin 1(*CAV1*), allograft inflammatory factor 1 (*AIF1*), ceruloplasmin (*CP*), lipocalin 2 (*LCN2*), complement component 1, s subcomponent (*C1S*), were significantly upregulated in the retinas during the early stages of an intraorbital nerve injury (Appendix 1). In addition, several genes involved in cell death, including macrophage cationic peptide 1 (*MCP-1*), TNF receptor superfamily member 6 (*Fas*), interferon induced with helicase C domain 1 (*IFIH1*), *BDNF*, *GADD45B*, and *B2M* were upregulated, suggesting that the damage to the optic nerve had already signaled to the soma of the RGCs and its surrounding extracellular matrix (Appendix 1). The mRNA expression levels of RGC-specific genes were lower than those of genes related to glial cells and cell death. The main reason may be the low relative frequency of RGCs in the retina [26] or it may indicate that axonal injury was not severe compared to the glial cells. A coupled two-way cluster of these differentially expressed genes between treated retinas and control retinas is shown in Figure 1. The highest significant genes (>2 fold change, p<0.05) are displayed in Appendix 2. They were then subjected to a Gene Ontology (GO) analysis. Appendix 3 shows the GO term categories with the biologic process, molecular function, and cellular component obtained from the GO annotation tool. This analysis revealed that seven of the genes were involved in the regulation of antigen processing and presentation (p=0.0224), 12 in the immune response (p=0.0016), and 14 in the immune system process (p=0.0020). All primary microarray data are available at the GEO website (GSE 29901).

Regulation of retinal gene expression after optic nerve injury is complex and controlled by various signaling pathways that regulate the balance between proliferation and differentiation. To examine whether there was recognizable biologic relevance to the gene’s expression patterns, a further functional pathway analysis was performed. The association between the significantly changed genes (p<0.05 and fold change >2) and the canonical pathway was determined based on a p value (p<0.05) calculated with Fisher’s exact test. Analysis revealed the most significant pathways included allograft rejection, antigen processing and presentation, cell adhesion molecules, complement and coagulation cascades, graft-versus-host disease, Type I diabetes mellitus, autoimmune thyroid disease, cytokine-cytokine receptor interaction, natural killer cell mediated cytotoxicity, RIG-I-like receptor signaling pathway, and the p53 signaling pathway. As shown in Table 2, the middle bar graph represents the p value of the disturbed signaling pathway. The genes altered in each pathway are listed on the right. No significant down-regulated ontology groups were found. GO and KEGG pathway database analyses provided a valuable mechanistic insight into retinas following an intraorbital nerve irradiated injury.

**Table 2:** Canonical pathway–based analyses of gene expression data in retinas, KEGG was used to identify the genes significantly associated with canonical pathways in the KEGG pathways database.

**Name**	**p value**	**Genes involved (p<0.05. Fold change>2)**
Allograft rejection	0	FAS, MAMU-A, MAMU-B18, MAMU-I
Antigen processing and presentation	0	B2M, MAMU-A, MAMU-B18, MAMU-I, LOC719379, LOC717726
Complement and coagulation cascades	0.0119	C1S, SERPING1
Graft-versus-host disease	0	FAS, MAMU-A, MAMU-B18, MAMU-I
P53 signaling pathway	0.001	DDB2, FAS, GADD45B,
Type I diabetes mellitus	0	FAS, MAMU-A, MAMU-B18, MAMU-I
Autoimmune thyroid disease	0.0004	FAS, MAMU-A, MAMU-I, MAMU-B18
Viral myocarditis	0.0028	MAMU-A, MAMU-B18, MAMU-I
Endocytosis	0.0021	MAMU-A, MAMU-B18, MAMU-I, PSD2
Cell adhesion molecules(CAMs)	0.0069	MAMU-A,MAMU-B18,MAMU-I, CLDN18
Natural killer cell mediated cytotoxicity	0.0007	FAS,MAMU-A,MAMU-B18,MAMU-I
Cytokine-cytokine receptor interaction	0.0004	CCL8,CXCL10, FAS,IL7,OSMR,MCP-1,CLDN18
Primary immunodeficiency	0.0041	BLNK, LOC717726
RIG-I-like receptor signaling pathway	0.0007	CXCL10, IFIH1,ISG15
NOD-like receptor signaling pathway	0.0016	CCL18, MCP-1
Chemokine signaling pathway	0.0128	CCL8, CXCL10, MCP-1
Peroxisome	0.0165	GNPAT, LOC574097
MAPK signaling pathway	0.0269	BDNF, FAS, GADD45B
Primary bile acid biosynthesis	0.0385	CH25H

Genes from the each treatment meeting the significance p value cutoff of 0.05 and fold change cutoff of 2 were associated with a canonical pathway in KEGG’s database, and considered for the analysis.

To validate microarray data, qRT–PCR analysis of mRNA expression changes was performed on five genes, which were chosen based on a combination of the array results and the potential biologic importance. The expression levels obtained by the analysis were normalized to that of 18S and are shown as the fold increase or decrease relative to that of the control. Table 3 shows the detailed qRT–PCR results. As seen from the results, when a gene-symbol only corresponds to one Probe Set-ID, the qRT–PCR analysis shows a similar pattern of mRNA expression changes in experimental and control retina as that shown by the microarray analysis; but when a gene symbol corresponds to more than one Probe Set-ID with a different change trend and call, the qRT–PCR results are not consistent with the microarray analysis. These genes filtered by the criteria (call value=P) are credible and worthy of further study.

**Table 3:** Confirmation of expression patterns of selected genes by quantitative RT–PCR.

		**Fold change in gene expression for monkeys (Microarray/qPCR) ON^a^**		
**GenBank accession number**	**Gene symbol**	**Monkey 01**	**Monkey 02**	**Monkey 03**
NM_001047137	***B2M***	2.66/2.83	3.8/2.22	2.35/3.4
XM_001089568	*BDNF*	2.02/1.59	3.05/0.83	1.71/2.33
XM_001098099	***GADD45B***	3.09/3.31	3.97/2.49	2.54/2.15
XM_001117266	*MDM2*	1.45/1.10	1.44/0.46	1.47/1.66
XM_001085609	*SCRG1*	2.93/0.93	12.45/0.20	46.79/1.26

^a^Fold change was used to evaluate retinal mRNA level by comparing samples of irradiated side with samples of control side. p values for the qPCR are at <0.05. ND, not determined. Genes only corresponding to one probe set-ID were shown in bold.

**Figure 2:**
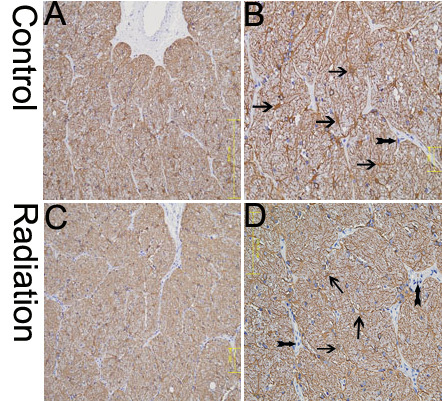
The comparative changes of glial fibrillary acidic protein (GFAP) immunohistochemical staining between control and treated optic nerve. The single arrows indicate astrocytes and the double arrows indicate inflammatory cells. **A**, **B**: The control, illustrating the typical bushy appearance of astrocytes with fine cellular processes. **C**, **D**: The astrocytic processes in the treated nerves displayed were shorter than those in the controls, were not straight, and were accompanied by decreased GFAP immunostaining. The images are representative of results obtained from three separate experiments. Scale bars: **A** and **C**, 200×; **B** and **D**; 400×.

**Figure 3:**
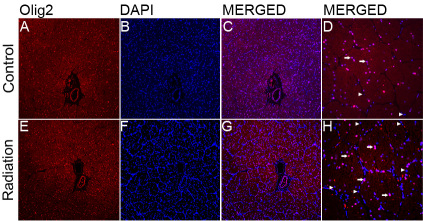
Double immunofluorescence staining for Olig2 (red) and DAPI (blue) on paraffin sections of the control and treated optic nerves using fluorescence microscopy. Overlay images demonstrate Olig2 and DAPI double positive cells. The cells positive for Olig2 (and not DAPI) are blood cells, and the cells positive for DAPI (and not Olig2) are inflammatory or microglial cells. The single arrows indicate oligodendrocyte cells, and the double arrows indicate inflammatory cells. The number of Olig2-positive cells and inflammatory cells apparently increased in the treated optic nerves. The images are representative of results obtained from three separate experiments. **A**–**D** control; **E**–**I** treated. Scale bars: **A**, **B**, **C**, **E**, **F**, and **H**; 100×; **D** and **I**; 400×.

**Figure 4:**
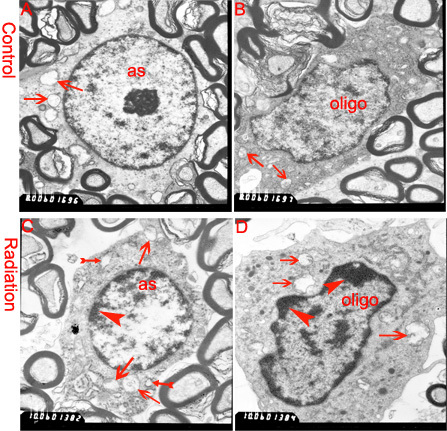
Electron micrographs of the astrocytes (**A**, **C**) and oligodendrocytes (**B**, **D**) of bilateral optic nerves. **A** and **B**, control; **C** and **D**, treated. Astrocytes (as); oligodendrocyte (oligo); swelling mitochondria (arrows); rough endoplasmic reticulum vacuoles (double arrows), and condensed chromatin (arrowheads). Scale bars: **C** and **D**;10,000×; **A** and **B**; 8,000×.

In addition, an optic nerve injury is regulated by a complex interplay between RGC-axons, astrocytes, and oligodendrocyte lineage cells. We observed the morphological changes between treated nerves (monkey #01 left ON, monkey #02 left ON, monkey #03 right ON) and control nerves (monkey #01 right ON, monkey #02 right ON, monkey #03 left ON). Light microscopy revealed that nerve fiber bundles in treated nerves were slightly edematous and irregularly arranged in a low magnification view of the optic nerve (Figure 2). High magnification showed more nucleated cells aggregated among nerve fiber bundles of the treated nerves than among the control nerves. Almost all of the astrocytic processes in the treated nerves were shorter than those in the controls, were not straight, and were accompanied by decreased GFAP immunostaining (Figure 2), which is a phenomenon referred to as “reactive gliosis.” Olig2-immunohistochemistry confirmed that most of the treated nerves were Olig2-positive oligodendrocytes and oligodendrocytic precursor cells. Others may be inflammatory cells or microglial cells (Figure 3). We did not detect a substantial alteration in expression of an endothelial cell marker CD34^+^ or axonal myelination in the bilateral optic nerves (data not shown). The cellular structures of the bilateral optic nerves were examined using the electron microscope, which depicted swollen mitochondria of different sizes in both treated and control nerves. Rough endoplasmic reticulum vacuoles and slight chromatin condensation were observed in treated nerves (Figure 4). Mitochondrial dysfunction leads to a severe decrease in ATP production, which impairs the function of glial cells and renders the optic nerve vulnerable to injury. Chromatin condensation along the nuclear membrane indicates early apoptosis of glial cells in the treated nerve, which began 24 h after the radiation injury to the intraorbital nerve.

## Discussion

An irradiated injury to an optic nerve will ultimately lead to radiation-induced optic neuropathy (RION), which is an irreversible process of visual function impairment. In this study, we explored genome-wide gene expression-associated alterations in bilateral retinas to observe early retinal, especially RGC, changes at the molecular level. We also examined morphological changes in the bilateral optic nerve using both light and electron microscopy, which might help illuminate the understanding of the pathogenesis of RION.

The microarray data revealed that a total of 1,597 genes met the criteria for differential expression and 82 genes (80 up, 2 down) met the criteria of a twofold increase or decrease and p<0.05. According to published microarray data [23-27], numerous RGC-specific genes in the retinas were induced by a radiation injury in the intraorbital nerve (Appendix 1). bHLHE40, a basic helix–loop–helix transcription factor, positioned genetically upstream of *Brn3b* [28-30], is involved in DNA-damage-induced apoptosis, which is an important regulator of cell differentiation, cell-cycle arrest, and apoptosis [31]. *Brn3b* is selectively expressed only in the retina in RGCs and serves as a central node in what is most certainly a highly complex gene regulatory network for RGC differentiation [25] and is essential for RGC differentiation, axonal outgrowth, and survival [32]. Deletion of *brn3b* results in short, disorganized RGC axons with dendritic rather than axonal features [25]. *STAT1* is frequently described as being proapoptotic and participates in the RGC degenerative pathway [33]. Upregulation of several key transcription factors implies that RGC death mechanisms were induced after a radiation injury by the astrocytic network [34] or injured axons. In addition to RGC-specific genes, other transcription factors of the same families might be related to RGC-specific genes because members of the same family may have similar roles.

In addition to RGC-specific genes, genes associated with glial activation were significantly activated in our study (Appendix 1). Activation of glial cells in the retina was considered a hallmark of neuronal damage [35], which may be involved in the initiation of RGC death [36]. This is consistent with previous reports. As a member of the lipocalin family, *Lcn2* is critical for cell death sensitization, stimulation of cell migration, and morphological changes in reactive astrocytes and it induces upregulation of *GFAP*, which is a marker for an astroglial reaction [37]. CAV1 is co-localized in hypertrophied GFAP-positive astrocytes with GFAP and participates in a variety of cellular functions, including signal translation [38], lipid metabolism [39], cell proliferation [40], and apoptotic cell death [41]. While activation of astrocytes (reactive gliosis) may inhibit neuroregeneration and neurite outgrowth [42], activated astrocytes may also provide a permissive substrate for axonal regrowth [43] or facilitate the integration of transplanted neural stem cells [44]. We speculate that axonal injury signals spread from the injured optic nerve to the surrounding Müller cells and other glial cells in the retina, and then results in their rapid activation.

Only the key genes that control cell cycle, DNA repair, and cell growth regulation were believed to be decisive in cell death or survival with the situation of lethal insults. In our study, several transcripts were upregulated more than fivefold. Among them, *MCP-1*, *FAS*, *BDNF*, *IFH1*, *B2M*, and *GADD45B* played a key role in the regulation of retinal cell survival and apoptosis. Generally, the *B2M* gene and *18S* rRNA have constant levels of expression across cell lines following exposure to ionizing radiation, thus they are considered as useful internal controls in gene expression analysis [45]. But the *B2M* gene expression levels were significantly elevated compared to control subjects in this study. The *B2M* gene encodes a serum protein found in association with the major histocompatibility complex (MHC) class I heavy chain on the surface of nearly all nucleated cells. The protein is potentially neurotoxic and can form amyloid fibrils in some pathological condition [46]. In clinical practice, the high concentration of *B2M* can be seen in hemodialysis patients. Because it cannot pass through the blood brain barrier (BBB), the central nervous system is not affected by amlyoid deposition. However, *B2M* in the central nervous system (CNS) induced by radiation treatment can persistently damage neuronal cells such as RGCs. *FAS* (CD95, APO-1) is a member of the TNF family of cytokine receptors, and a potent inducer of apoptosis [47]. In our study, *FAS* gene expression in the retinas of irradiated nerves was upregulated. *FAS*-mediated apoptosis also participates in the disposition of radiation-injured nerves in vivo. In addition, there is limited early stage survival signaling in the treated retina following an optic nerve injury. *GADD45B* is a key member of nuclear proteins, is inducible by DNA-damaging stresses, and has been shown to play an active role in cell cycle arrest, apoptosis, and signal transduction that affects cell survival. It has long been observed that IR-induced *GADD45B* expression linked to *p53* pathways as well as mitogen-activated protein kinases (MAPKs) pathways are important components of the intrinsic neuroprotective mechanisms of RGC neurons in the retina [48]. *BDNF* is an important trophic factor for RGC cells and has been shown to be neuroprotective in RGC injury paradigms. However, the upregulation of insulin-like growth factor (*IGF*)*-1* and related factors antagonizes the trophic activity of *BDNF* [49].

Our microarray data also showed upregulation of *IL7*, *IL1B*, *IL8RB*, *MMP2*, *CCL8*, *CXCL10*, and *TNFRSF11B*, with downregulation of *IL2*, and *IL5* among different genes. *IL-7* and *IL-2* are members of the γ–chain family of cytokines that have been implicated in the process of memory CD8+ T cell generation [50,51]. *IL-7* was believed to be crucial for providing survival signals to naive and memory CD8+ T cells [52]. Moreover, a previous study has demonstrated that *IL-2* downregulated *IL-7Ra* expression in activated T cells. The cytotoxic function of these antigen-specific CD8+ T cells has been reported to play an important role in keeping chronic infection under control. *IL1B* is a potent pro-inflammatory cytokine that is primarily produced by microglia in the brain and it plays a key role in the pathogenesis of several acute and chronic neurologic disorders. The above changes indicate that an acute inflammatory response in the treated retinas occurred as early as in the first 24 h after a radiation injury to the intraorbital nerve, which could change normal communication between cells and contribute to additional secondary damage from the ongoing inflammatory responses.

Regulation of gene expression is complex and controlled by various signaling pathways. These signals play critical roles in controlling cell survival and repopulation effects following irradiation, in a cell-type-dependent manner. That is to say, most of these altered genes do not necessarily produce the final biologic effects, but regulate other genes. So in this study, we employed both GO analysis with GenMAPP and KEGG pathway annotation. Using these integrated tools, we identified significant signal pathways based on *B2M* and *GADD45B* (p<0.05). Analysis of pathways revealed the most significant pathways included antigen processing and presentation, the MAPK signaling pathway, cell cycle, and the *p53* signaling pathway.

Expression of the GADD (growth arrest and DNA damage) gene family occurs with most conditions inducing DNA damage or growth arrest. Induction of *GADD45* mRNA and *GADD45* protein expression depends on a wild-type p53 phenotype and p53-dependent G1 cell cycle arrest [53], suggesting that *GADD45* may function as a downstream effector for *p53* in the radiation-induced G1 delay [54]. We have shown here that *p53*, *MDM2*, BCL2-associated X protein (*BAX*), *GADD45B*, and TP53 apoptosis effector (*PERP*) in the retina are upregulated in response to radiation treatment of the intraorbital nerve. These results suggest that radiation treatment of the intraorbital nerve led to retinal cell DNA damage, and modulation in the expression of the tumor suppressor’s *p53*/*p21* signaling pathway plays an important role in the development of vision loss after radiation.

Apart from the *P53* tumor suppressor pathway, we also found that the three major MAPK signaling pathways—p38 MAPK, extracellular signal-regulated protein kinases, and c-Jun N-terminal protein kinase—were activated in our study. An increasing body of evidence suggests that MAPKs play a key role in transmitting cellular stress stimuli, such as ischemia, heat shock, microbial infection, and irradiation by sequential protein phosphorylation [55,56]. The p38 MAPK pathway, in a manner not dissimilar to that described for the JNK pathway, plays an important role in the control of radiation-induced G2/M arrest, which is protective, and, in certain cell types, may also play a proapoptotic role via the induction of GADD transcription factors. Activation of the JNK pathway was initially linked to the toxic effects of radiation signaling [39]. Recent studies have reported that the p38 MAPK signaling pathway in several different inflammatory models regulates vascular inflammation and epithelial barrier dysfunction in the formation of proinflammatory cytokines, such as tumor necrosis factor α, interleukin (IL) 6, and the CXC chemokine *IL-8* [57].

Astroglia and oligodendroglia are the two most common types of glia in the optic nerve and constitute the large majority of optic nerve cells. The astrocytic network encapsulates the microvessel wall with perivascular endfeet, provides structural, trophic, and metabolic support and maintains synaptic functions by buffering ion concentrations, clearing released neurotransmitters [58]. Damage to astrocytes will inevitably break down the functioning of the astrocytic network and BBB, which will lead to metabolic disorders in oligodendrocytes, vascular enthothelial cells, other glial cells, and reactive gliosis. Oligodendrocytes, which are more sensitive to injury induced by specific stimuli and are much more susceptible to cell death than astrocytes and neuronal cells [59], were significantly increased in the treated nerves compared with the control ones (mean±SD: 334±35 cells/field versus 265±11 cells/field, p<0.05). The increased oligodendrocytes evoke neuronal repair from oligodendrocytes precursor cells and provide trophic support after a nerve injury. But differentiating oligodendroglial cells are especially sensitive to mitochondrial dysfunction, and even a slight injury will completely interrupt the differentiation program [60]. Worse, the increase in oligodendrocyte precursor cells will inevitably exacerbate metabolic insults for this condition. Our study showed swelling of the mitochondria in bilateral optic nerves, which lead to a severe decrease in ATP production, rendering the optic nerve vulnerable to injury and repressing proliferation of differentiating oligodendroglial cells.

The major drawback of this study is that there were no control groups administered with different doses, primarily because of the limited number of experimental animals. We took measures to reduce the radiation dose to bilateral eyeballs and the contralateral optic nerve. The ipsilateral eyeball reached 3 Gy, the contralateral eyeball reached 1 Gy. Whether retinal damage was caused directly by this low dose or indirectly by intraorbital nerve irradiation injury needs to be studied further. Another drawback was that our samples contained the whole neural retina, which is composed of many cell types. RGCs were only a small part of the retina. Studies of dissociated, cultured RGCs indicate gene expression unique to RGCs, but under these conditions, their axons were transected and they were separated from their normal condition. The real changes and interactions among retinal cell types could not be reflected in vivo.

The mammalian retina is a complex tissue composed of neuronal, glial, and vascular cell types among which RGCs are the only type of output neurons that send their axons to the visual cortex of the brain. Similar to immature motor neurons, adult mammalian retinal ganglion cells are particularly vulnerable to the crushing or transaction of their axons in the optic nerve, the loss of which is irreversible. Damage to axons can signal the soma of the RGCs in the retina within 30 min of an axonal injury, which then initiates pathways of cell death in RGCs within 6 h of the injury. Although RGC degeneration and damage are present in glaucoma and other ocular neuropathies, little is known about the retinal reactions at the genetic and molecular levels, especially regarding RGC and glial reactions in the retina, in response to a radiation injury to the intraorbital nerve. We hypothesize that an up-threshold radiation injury to the optic nerve is capable of triggering RION. Our microarray results demonstrate that the current radiation dose damaged RGCs in the retina, and genes associated with glial cells in the retina were more sensitive than RGC-specific genes in response to a radiation injury to the intraorbital nerve. Morphological changes in bilateral optic nerves show that the primary insult to the optic nerve after radiation treatment possibly does not lie in myelinated axons but in the glial cells in the optic nerve. We envision that one of the several types of glial cells rather than intrinsic neurons initiates the optic nerve injury after radiation treatment. The dysfunction will lead to glial cell death or to a fibroglial reaction. Furthermore, injury signals from the optic nerves also signal the retina through the astrocytic network. Such effects will break down BBB and ultimately lead to dymyelination of the optic nerve, reactive astrocytosis, and the death of vascular endothelial cells and RGCs.

## Appendix 1. Candidate optic neuropathy genes expressed by retina.

To access the data, click or select the words “Appendix 1.” This will initiate the download of a compressed (pdf) archive that contains the file.

## Appendix 2. Genes with fold change>2 and p<0.05 in retina after radiated injury of intraorbital optic nerve.

To access the data, click or select the words “Appendix 2.” This will initiate the download of a compressed (pdf) archive that contains the file. FC refers to fold up- or down- regulation of each gene expressed in treated retinas in relation to expression in untreated retinas. The p values for these genes are all less than 0.05.

## Appendix 3. Gene expression profiles in the retina after optic nerve irradiated injury.

To access the data, click or select the words “Appendix 3.” This will initiate the download of a compressed (pdf) archive that contains the file.

## References

[1] Dieckmann K, Georg D, Zehetmayer M, Bogner J, Georgopoulos M, Pötter R (2003). LINAC based stereotactic radiotherapy of uveal melanoma: 4 years clinical experience.. Radiother Oncol.

[2] Zabramski JM, Kiris T, Sankhla SK, Cabiol J, Spetzler RF (1998). Orbitozygomatic craniotomy. Technical note.. J Neurosurg.

[3] Klink DF, Miller NR, Williams J (1998). Preservation of residual vision 2 years after stereotactic radiosurgery for a presumed optic nerve sheath meningioma.. J Neuroophthalmol.

[4] Thompson TP, Lunsford LD, Flickinger JC (2000). Radiosurgery for hemangiomas of the cavernous sinus and orbit: Technical case report.. Neurosurgery.

[5] Yang Z, Quigley HA, Pease ME, Yang Y, Qian J, Valenta D, Zack DJ (2007). Changes in gene expression in experimental glaucoma and optic nerve transection: the equilibrium between protective and detrimental mechanisms.. Invest Ophthalmol Vis Sci.

[6] Agudo M, Pérez-Marín MC, Lönngren U, Sobrado P, Conesa A, Cánovas I, Salinas-Navarro M, Miralles-Imperial J, Hallböök F, Vidal-Sanz M (2008). Time course profiling of the retinal transcriptome after optic nerve transection and optic nerve crush.. Mol Vis.

[7] Wang AG, Chen CH, Yang CW, Yen MY, Hsu WM, Liu JH, Fann MJ (2002). Change of gene expression profiles in the retina following optic nerve injury.. Brain Res Mol Brain Res.

[8] Kim M-S, Park K, Kim JH, Kim Y-D, Lee JI (2008). Gamma knife radiosurgery for orbital tumors.. Clin Neurol Neurosurg.

[9] Leber KA, Berglof J, Pendl G (1998). Dose-response of the visual pathways and cranial nerves of the cavernous sinus to stereotactic radiosurgery.. J Neurosurg.

[10] Kruse JJ, te Poele JA, Velds A, Kerkhoven RM, Boersma LJ, Russell NS, Stewart FA (2004). Identification of differentially expressed genes in mouse kidney after irradiation using microarray analysis.. Radiat Res.

[11] Lehnert S (2000). Prediction of tumor response to therapy: molecular markers and the microenvironment. Apoptosis and chips: An overview of the proceedings.. Radiat Res.

[12] Tada M, Matsumoto R, Iggo RD, Onimaru R, Shirato H, Sawamura Y, Shinohe Y (1998). Selective sensitivity to radiation of cerebral glioblastomas harboring p53 mutations.. Cancer Res.

[13] Joki T, Carroll RS, Dunn IF, Zhang J, Abe T, Black PM (2001). Assessment of alterations in gene expression in recurrent malignant glioma after radiotherapy using complementary deoxyribonucleic acid microarrays.. Neurosurgery.

[14] Kang CM, Cho HN, Ahn JM, Lee SS, Jeoung DI, Cho CK, Bae S, Lee SJ, Lee YS (2004). Alteration of gene expression during radiation-induced resistance and tumorigenesis in NIH3T3 cells revealed by cDNA microarrays: involvement of MDM2 and CDC25B.. Carcinogenesis.

[15] Rosen EM, Fan S, Rockwell S, Goldberg ID (1999). The molecular and cellular basis of radiosensitivity: Implications for understanding how normal tissues and tumors respond to therapeutic radiation.. Cancer Invest.

[16] Fornace AJ, Amundson SA, Bittner M, Myers TG, Meltzer P, Weinsten JN, Trent J (1999). The complexity of radiation stress responses: Analysis by informatics and functional genomics approaches.. Gene Expr.

[17] Kubota N, Okada S, Inada T, Ohnishi K, Ohnishi T (2000). Wortmannin sensitizes human glioblastoma cell lines carrying mutant and wild type TP53 gene to radiation.. Cancer Lett.

[18] Lammering G, Lin PS, Contessa JN, Hampton JL, Valerie K, Schmidt-Ullrich RK (2001). Adenovirus-mediated overexpression of dominant negative epidermal growth factor receptor-CD533 as a gene therapeutic approach radiosensitizes human carcinoma and malignant glioma cells.. Int J Radiat Oncol Biol Phys.

[19] Chinnaiyan P, Huang S, Vallabhaneni G, Armstrong E, Varambally S, Tomlins SA, Chinnaiyan AM, Harari PM (2005). Mechanisms of enhanced radiation response following epidermal growth factor receptor signaling inhibition by erlotinib (Tarceva). Cancer Res.

[20] Iversen LL, Bloom FE (1972). Studies of the uptake of 3H-GABA and [3H] glycine in slices and homogenates of rat brain and spinal cord by electron microscopic autoradiography.. Brain Res.

[21] Glowinski J, Iversen LL (1966). Regional studies of catecholamines in the rat brain. I. The disposition of [3H] norepinephrine. J Neurochem.

[22] Li YQ, Chen P, Haimovitz-Friedman A, Reilly RM, Wong CS (2003). Endothelial apoptosis initiates acute blood-brain barrier disruption after ionizing radiation.. Cancer Res.

[23] Ivanov D, Dvoriantchikova G, Nathanson L, McKinnon SJ, Shestopalov VI (2006). Microarray analysis of gene expression in adult retinal ganglion cells.. FEBS Lett.

[24] Guo Y, Johnson EC, Cepurna WO, Dyck JA, Doser T, Morrison JC (2011). Early Gene Expression changes in the retinal ganglion cell layer of a rat glaucoma model.. Invest Ophthalmol Vis Sci.

[25] Mu X, Klein WH (2004). A gene regulatory hierarchy for retinal ganglion cell specification and differentiation.. Semin Cell Dev Biol.

[26] Farkas RH, Qian J, Goldberg JL, Quigley HA, Zack DJ (2004). Gene Expression Profiling of purified rat retinal ganglion cells.. Invest Ophthalmol Vis Sci.

[27] Kim CY, Kuehn MH, Clark AF, Kwon YH (2006). Gene expression profile of the adult human retinal ganglion cell layer.. Mol Vis.

[28] Brown NL, Kanekar S, Vetter ML, Tucker PK, Gemza DL, Glaser T (1998). Math5 encodes a murine basic helix-loop-helix transcription factor expressed during early stages of retinal neurogenesis.. Development.

[29] Brown NL, Patel S, Brzezinski J, Glaser T (2001). Math5 is required for retinal ganglion cell and optic nerve formation.. Development.

[30] Wang SW, Kim BS, Ding K, Wang H, Sun D, Johnson RL, Klein WH, Gan L (2001). Requirement for math5 in the development of retinal ganglion cells.. Genes Dev.

[31] Thin TH, Li L, Chung TK, Sun H, Taneja R (2007). Stra13 is induced by genotoxic stress and regulates ionizing-radiation-induced apoptosis.. EMBO Rep.

[32] Mu X, Beremand PD, Zhao S, Pershad R, Sun H, Scarpa A, Liang S, Thomas TL, Klein WH (2004). Discrete gene sets depend on POU domain transcription factor Brn3b/Brn-3.2/POU4f2 for their expression in the mouse embryonic retina.. Development.

[33] Samardzija M, Wenzel A, Aufenberg S, Thiersch M, Remé C, Grimm C (2006). Differential role of Jak-STAT signaling in retinal degenerations.. FASEB J.

[34] Fitzgerald M, Bartlett CA, Harvey AR, Dunlop SA (2010). Early Events of Secondary Degeneration after partial optic nerve transection: An immunohistochemical study.. J Neurotrauma.

[35] Zhang S, Wang H, Lu Q, Qing G, Wang N, Wang Y, Li S, Yang D, Yan F (2009). Detection of early neuron degeneration and accompanying glial responses in the visual pathway in a rat model of acute intraocular hypertension.. Brain Res.

[36] Zhang X, Cheng M, Chintala SK (2004). Optic nerve ligation leads to astrocyte-associated matrix metalloproteinase-9induction in the mouse retina.. Neurosci Lett.

[37] Lee S, Park JY, Lee WH, Kim H, Park HC, Mori K, Suk K (2009). Lipocalin-2 Is an Autocrine mediator of reactive astrocytosis.. J Neurosci.

[38] Cameron PL, Liu C, Smart DK, Hantus ST, Fick JR, Cameron RS (2002). Caveolin-1 expression is maintained in rat and human astroglioma cell lines.. Glia.

[39] Ikezu T, Ueda H, Trapp BD, Nishiyama K, Sha JF, Volonte D, Galbiati F, Byrd AL, Bassell G, Serizawa H, Lane WS, Lisanti MP, Okamoto T (1998). Affinity-purification and characterization of caveolins from the brain:Differential expression of caveolin-1, −2, and −3 in brain endothelial and astroglial cell types.. Brain Res.

[40] Okamoto T, Schlegel A, Scherer PE, Lisanti MP (1998). Caveolins, a family of scaffolding proteins for organizing “preassembled signaling complexes” at the plasma membrane.. J Biol Chem.

[41] Williams TM, Lisanti MP (2004). The Caveolin genes: From cell biology to medicine.. Ann Med.

[42] Silver J, Miller JH (2004). Regeneration beyond the glial scar.. Nat Rev Neurosci.

[43] Ridet JL, Malhotra SK, Privat A, Gage FH (1997). Reactive astrocytes: Cellular and molecular cues to biological function.. Trends Neurosci.

[44] Nishida A, Takahashi M, Tanihara H, Nakano I, Takahashi JB, Mizoguchi A, Ide C, Honda Y (2000). Incorporation and differentiation of hippocampus-derived neural stem cells transplanted in injured adult rat retina.. Invest Ophthalmol Vis Sci.

[45] Banda M, Bommineni A, Thomas RA, Luckinbill LS, Tucker JD (2008). Evaluation and validation of housekeeping genes in response to ionizing radiation and chemical exposure for normalizing RNA expression in real-time PCR.. Mutat Res.

[46] Giorgetti S, Raimondi S, Cassinelli S, Bucciantini M, Stefani M, Gregorini G, Albonico G, Moratti R, Montagna G, Stoppini M, Bellotti V (2009). β2-Microglobulin is potentially neurotoxic, but the blood brain barrier is likely to protect the brain from its toxicity.. Nephrol Dial Transplant.

[47] Locksley RM, Killeen N, Lenardo MJ (2001). The TNF and TNF receptor superfamilies: Integrating mammalian biology.. Cell.

[48] Liu B, Suyeoka G, Papa S, Franzoso G, Neufeld AH (2009). Growth arrest and DNA damage protein 45b (Gadd45b) protects retinal ganglion cells from injuries.. Neurobiol Dis.

[49] Fan W, Agarwal N, Cooper NG (2006). The role of CaMKII in BDNF-mediated neuroprotection of retinal ganglion cells (RGC-5).. Brain Res.

[50] Schluns KS, Lefrancois L (2003). Cytokine control of memory T-cell development and survival.. Nat Rev Immunol.

[51] Prlic M, Lefrancois L, Jameson SC (2002). Multiple choices: Regulation of memory CD8 T cell generation and homeostasis by interleukin (IL)-7 and IL-15.. J Exp Med.

[52] Ma A, Koka R, Burkett P (2006). Diverse functions of IL-2, IL-15, and IL-7 in lymphoid homeostasis.. Annu Rev Immunol.

[53] Kastan MB, Zhan Q, el-Deiry WS, Carrier F, Jacks T, Walsh WV, Plunkett BS, Vogelstein B, Fornace AJ (1992). A mammalian cell cycle checkpoint pathway utilizing p53 and GADD45 is defective in ataxia-telangiectasia.. Cell.

[54] Zhan Q, Bae I, Kastan MB, Fornace AJJ (1994). The p53-dependent gamma-ray response of GADD45.. Cancer Res.

[55] Dent P, Yacoub A, Fisher PB, Hagan MP, Grant S (2003). MAPK pathways in radiation responses.. Oncogene.

[56] Kumar S, Boehm J, Lee JC (2003). P38 MAP kinases: Key signaling molecules as therapeutic targets for inflammatory diseases.. Nat Rev Drug Discov.

[57] Mihaescu A, Santen S, Jeppsson B, Thorlacius H (2010). P38 Mitogen-activated protein kinase signaling regulates vascular inflammation and epithelial barrier dysfunction in an experimental model of radiation-induced colitis.. Br J Surg.

[58] Baumann N, Pham-Dinh D (2001). Biology of oligodendrocyte and myelin in the mammalian central nervous system.. Physiol Rev.

[59] Casaccia-Bonnefil P, Aibel L, Chao MV (1996). Central glial and neuronal populations display differential sensitivity to ceramide-dependent cell death.. J Neurosci Res.

[60] Schoenfeld R, Wong A, Silva J, Li M, Itoh A, Horiuchi M, Itoh T, Pleasure D, Cortopassi G (2010). Oligodendroglial differentiation induces mitochondrial genes and inhibition of mitochondrial function represses oligodendroglial differentiation.. Mitochondrion.

